# Can cardiovascular risk management be improved by shared care with general practice to prevent cognitive decline following stroke/TIA? A feasibility randomised controlled trial (SERVED memory)

**DOI:** 10.1186/s12877-020-01760-z

**Published:** 2020-09-17

**Authors:** William J. Davison, Phyo K. Myint, Yoon K. Loke, Garth Ravenhill, David Turner, Chris Fox, Lee Shepstone, John F. Potter

**Affiliations:** 1 Ageing and Stroke Medicine, Norwich Medical School, University of East Anglia, Bob Champion Research Building, James Watson Road, Norwich, UK; 2 Ageing Clinical & Experimental Research Team (ACER), Institute of Applied Health Sciences, University of Aberdeen, Aberdeen, UK; 3 Norwich Medical School, University of East Anglia, Norwich, UK; 4 Norfolk and Norwich University Hospital, Norwich, UK

**Keywords:** Cognitive impairment, Dementia after stroke, Vascular dementia, Stroke, Cerebrovascular disease

## Abstract

**Background:**

Cognitive impairment and dementia following cerebrovascular disease are increasingly common in the UK. One potential strategy to prevent post-stroke cognitive decline is multimodal vascular risk factor management. However, its efficacy remains uncertain and its application in vulnerable patients with incident cerebrovascular disease and early cognitive impairment has not been assessed.

The primary aim of this study was to assess the feasibility of recruitment and retention of patients with early cognitive impairment post-stroke or transient ischaemic attack (TIA) to a trial of enhanced vascular risk factor management combining primary and secondary care.

**Methods:**

In this single centre, open label trial adults with a recent stroke or TIA and mild cognitive impairment (MCI) were randomised 1:1 to a three-monthly multimodal vascular risk factor intervention jointly delivered by the trial team and General Practitioner (GP), or control (defined as usual care from the GP). Chosen risk factors were blood pressure (BP), total cholesterol, blood glucose (HbA1C) in those with diabetes, and heart rate and adequacy of anticoagulation in those with atrial fibrillation (AF). Similar patients with normal cognition were enrolled in an embedded observational cohort and also received usual care from the GP. Repeat cognitive screening was undertaken in all participants after 12 months.

**Results:**

Seventy three participants were recruited to the randomised trial and 94 to the observational cohort (21.8% of those screened). From the randomised trial 35/73 (47.9%) dropped out before final follow-up. In all groups guideline based rates of risk factor control were mostly poor at baseline and did not significantly improve during follow-up. The observational cohort demonstrated greater decline in cognitive test scores at 12 months, with no difference between the randomised groups.

**Conclusions:**

Recruitment to such a study was feasible, but retention of participants was difficult and generally poor rates of risk factor control suggested insufficient application of the intervention. Consequently, successful scaling up of the trial would require protocol changes with less reliance on primary care services. Any future trial should include participants with normal cognition post-stroke as they may be at greatest risk of cognitive decline.

**Trial registration:**

ISRCTN, ISRCTN42688361. Registered 16 April 2015.

## Background

Dementia is a significant and increasing health problem in the UK, yet disease modifying treatments are lacking [1], therefore strategies to prevent cognitive decline are desirable. Given that cognitive impairment may affect up to 40% of patients following stroke and TIA [2–4], such strategies may be particularly valuable in this patient group. One potential strategy is multimodal vascular risk factor control as these risk factors contribute to recurrent stroke as well as both vascular dementia (VaD) and Alzheimer’s disease [5–7], and their presence also increases the risk of early cognitive decline progressing to dementia [8]. Evidence supports the value of good BP control for reducing the risk of subsequent severe cognitive impairment post-stroke, yet there remains uncertainty about the value of targeting other vascular risk factors that are relevant to secondary stroke recurrence, especially as part of a multimodal risk factor approach [5, 9, 10]. Furthermore, whether targeting such a strategy at patients who already have MCI post-stroke in order to prevent further cognitive deterioration has not been studied [11–13].

SERVED Memory (Screening and Enhanced Risk factor management to prevent Vascular Event related Decline in Memory) was developed to investigate the feasibility of recruiting patients with MCI post-stroke or TIA to a pragmatic intervention trial of enhanced vascular risk factor management. It was hypothesised that enhanced risk factor management with a “treat to target” approach, delivered by a combination of the patient’s GP and a trial team, would be safe and effective, potentially reducing the risk of progression of MCI compared to standard GP management alone. The trial also incorporated an embedded non-randomised observational cohort with the aim of providing epidemiological data regarding the natural history of cognitive impairment post-stroke or TIA.

## Methods

SERVED Memory was a single-centre, open-label parallel group randomised controlled feasibility trial, with embedded non-randomised observational cohort. The trial was granted ethical approval and was prospectively registered (ISRCTN 42688361). The full trial protocol has previously been published [14]. This report adheres to the CONSORT guidelines for pilot and feasibility trials.

In brief, participants were recruited from stroke services at the Norfolk and Norwich University Hospital (NNUH). Adults with a mild stroke or TIA within the last 8 weeks and Montreal Cognitive Assessment (MoCA) score ≥ 26 were eligible for the observational cohort, and those with a MoCA score consistent with MCI (i.e. 20–25 [11, 12]) were eligible for the randomised controlled trial (RCT). Patients with life expectancy < 1 year, diagnosed depression, or MoCA score < 20 were excluded. All participants provided written informed consent. RCT participants were randomised 1:1 by computer generated randomisation table, with block size of four, to an intervention or control group. Enrolment, randomisation, and intervention allocation were undertaken by the trial nurses or research fellow. Baseline recording of demographic data, medication use and compliance, and vascular risk factors was completed. Measured risk factors were clinic BP, total cholesterol, blood glucose HbA1c in those with diabetes, and heart rate and anticoagulation adequacy for those with AF. Targets were ideal BP < 130/80 mmHg and standard < 140/90 mmHg [15, 16]; total cholesterol < 4.0 mmol/L (non-fasting); HbA1C 48-53 mmol/mol; heart rate 60–80 beats per minute for those in AF. Adequate anticoagulation was defined as taking warfarin with INR 2.5–3.0, or a direct oral anticoagulant, unless contraindicated. Observation and control participants received usual care from their GP only. Intervention participants were seen in hospital by the trial team at 3, 6, and 9 months post-randomisation for risk factor assessment. Results were passed immediately to the GP for action by phone and letter with the trial team only making treatment alterations when necessary for patient safety. All participants were followed up at 12 months for assessment of risk factors, medication adherence, adverse events and repeat MoCA. Baseline frailty was retrospectively assessed from clinical notes using the Rockwood Frailty Score by a stroke physician blinded to group allocation.

The primary outcome was the assessment of rates of recruitment and retention at 12 months from screening and management logs. Secondary outcomes were (i) rates of risk factor control to the specified targets in each group (ii) differences in the change in MoCA score between the intervention and control groups, (iii) change in MoCA score in the observational arm, and (iv) rates of adverse events (including recurrent stroke) in each group.

A convenience sample size was based on estimates of the prevalence of cognitive impairment in patients with incident stroke/TIA [4], the incidence of dementia post-stroke [17], and estimated cognitive screening rates at NNUH [4]. Based on these estimates target numbers were 100 in the RCT (50 per group) and 100 in the observational cohort.

### Statistical analysis

Data were analysed using SPSS (version 25.0) with descriptive statistics only unless specified. Screening logs were assessed to determine the proportion of eligible participants who consented to participate in the trial, including the proportion that would have been eligible for the RCT. Management logs were assessed for retention rates in each trial arm and, where possible, reasons for attrition were identified. Proportions of participants with controlled risk factors in each group were calculated at baseline and follow-up along with the frequency of medication changes that occurred during the trial. Changes in MoCA score from baseline to follow-up for each arm were assessed using a paired samples t test, with further testing of any difference between the intervention and control arms. A general linear model, with a normal error term, was used to estimate the effect of the intervention, with a 95% confidence interval, on the 12 month MoCA values. The model included randomisation group (intervention or control), sex, diagnosis (stroke or TIA) and baseline MoCA value. Post-hoc analysis of the difference in baseline frailty score in retained vs. not retained participants was assessed with a Mann-Whitney U test.

## Results

**Fig. 1:**
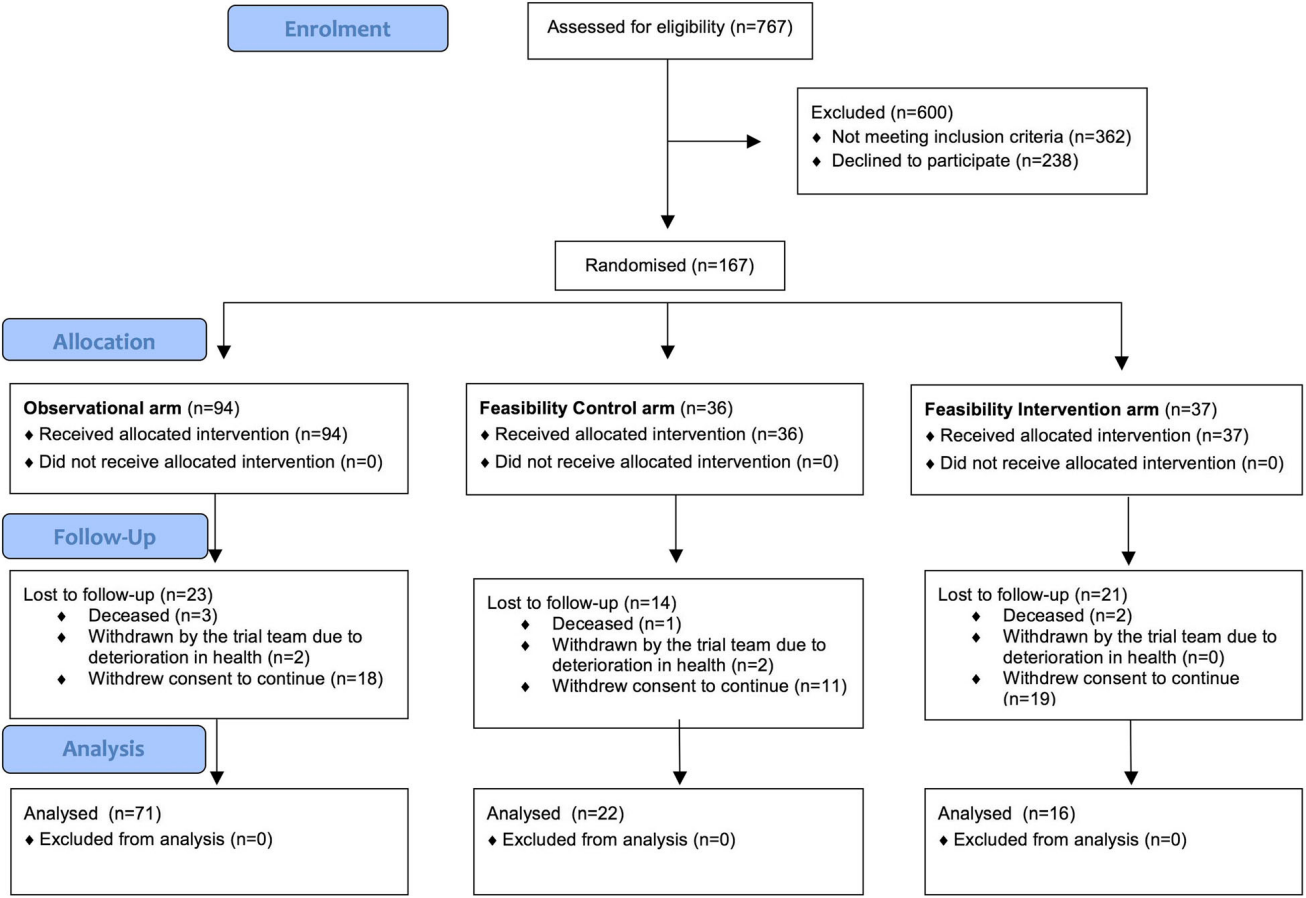
CONSORT flow diagram

Trial recruitment ran from November 2015 to July 2017, with final follow-up completed 12 months later. This was the pre-planned trial duration. Seven hundred and sixty-seven patients were screened, with 167 (21.8%) providing consent to participate (Fig. 1). Ninety-four participants were included in the observational cohort and 73 were allocated to the RCT, 37 being randomised to intervention and 36 to control. Of the remainder screened 362 (47.2%) patients were ineligible and 238 (31.0%) were eligible but declined to participate. Of those declining to participate 18/238 (7.6%) had a MoCA score ≥ 26, 50/238 (21.0%) had a MoCA score between 20 and 25, and 170/238 (71.4%) had not completed cognitive testing at the time of screening. Demographic details are presented in Table 1. Both randomised groups were well matched in most areas.

**Table 1:** Demographic data for each group at baseline

		Observation	Control	Intervention
**N**		94	36	37
**Symptom onset to randomisation (days)**		25.7 (20.1)	22.6 (20.9)	17.8 (19.7)
**Age (years)**		72.1 (10.9)	74.9 (9.2)	75.0 (12.0)
**Sex (male)**		59 (62.8%)	23 (63.9%)	27 (73.0%)
**Ethnicity (White British)**		94 (100.0%)	36 (100.0%)	37 (100.0%)
**Smoking status**	**Non-smoker**	38 (40.4%)	17 (47.2%)	26 (70.3%)
	**Ex-smoker**	29 (30.9%)	14 (38.9%)	10 (27.0%)
	**Current smoker**	6 (6.4%)	5 (13.9%)	1 (2.7%)
**Alcohol (units/wk)**		0.0 (0.0, 15.8)	3.0 (0.0, 20.0)	2.0 (0.0, 9.0)
**Diagnosis**	**TIA**	40 (42.6%)	11 (30.6%)	10 (27.0%)
	**Stroke**	54 (57.4%)	25 (69.4%)	27 (73.0%)
**OCSP classification**	**LACS**	27 (50.0%)	9 (36.0%)	11 (40.7%)
	**PACS**	13 (24.1%)	13 (52.0%)	11 (40.7%)
	**TACS**	0 (0.0%)	0 (0.0%)	0 (0.0%)
	**POCS**	14 (25.9%)	3 (12.0%)	5 (18.5)
**Past medical history**	**AF**	25 (26.6%)	6 (16.7%)	10 (27.0%)
	**Diabetes**	19 (20.2%)	7 (19.4%)	5 (13.5%)
	**IHD**	11 (11.7%)	4 (11.1%)	6 (16.2%)
	**Stroke**	44 (46.8%)	12 (33.3%)	21 (56.8%)
	**TIA**	36 (38.3%)	6 (16.7%)	7 (18.9%)
	**Hypertension**	53 (56.4%)	20 (55.6%)	25 (67.6%)
**Rockwood Frailty Score**		4.0 (3.0, 6.0)	5.0 (4.0, 6.0)	6.0 (4.5, 6.0)
**MoCA**		27.4 (1.4)	23.4 (1.4)	23.2 (1.5)
**Clinic BP (mmHg)**	**Systolic**	147.3 (20.5)	148.1 (21.0)	145.2 (19.5)
	**Diastolic**	79.6 (10.5)	78.9 (11.5)	81.8 (12.5)
**Total Cholesterol (mmol/L)**		4.9 (1.2)	4.9 (1.2)	4.6 (1.4)
**Heart rate (beats per min)**^**a**^		76.6 (18.9)	75.9 (16.8)	80.4 (10.2)
**On anticoagulation**^**a**^		10/25 (40.0%)	3/6 (50.0%)	3/10 (30.0%)
**HbA1C (mmol/mol)**^**b**^		52.5 (47.3, 69.5)	49.5 (43.0, 82.3)	73.0 (51.8, 106.3)

Data presented are mean (SD), median (IQR), or frequency (%)

^a^Only those with AF

^b^Only those with diabetes

During follow-up 35/73 (47.9%) randomised participants withdrew (25 [71.4%]) or were lost to follow-up (10 [28.6%]), 14/36 (38.9%) from the control group and 21/37 (56.8%) from the intervention group. The majority of participants (29/35 [82.9%]) not completing the trial withdrew consent to continue. Of the remainder, three died and three were withdrawn by the trial team due to a deterioration in health. Participants were not required to provide a reason for dropping out, but recurring reasons offered included ‘deteriorating health’ and ‘not wishing to travel to the hospital for follow-up visits’.

Average MoCA scores declined significantly in the observation cohort (− 1.7 points [95%CI − 2.3 to − 1.1, *p* < 0.0001]), but not in the intervention (− 0.6 points [95%CI − 2.3 to 1.1, *p* = 0.45]) or control groups (− 0.5 points [95%CI − 2.1 to 1.1, p = 0.45]). From the general linear model to estimate the effect of the intervention the mean 12 month MoCA for the Intervention group was 0.664 units lower than for Control, with 95% confidence interval for the difference (intervention minus control) being − 2.69 to 1.37. Baseline rates of control for all risk factors were low across all trial groups, irrespective of BP threshold value (Table 2 and Additional file 1). There were improvements in the rates of control for cholesterol and adequate anticoagulation in all trial groups at 12 months, however, BP control rates had declined and no changes were seen in relation to heart rate and HbA1C (Additional file 2). The proportions of participants on treatment for the selected risk factors were largely unaltered after 12 months, with the exception of increases in statin use and the prescription of anticoagulants. Rates of adverse events and recurrent stroke were similar between the randomised groups (Additional file 3). Median baseline frailty scores were lower in those who completed the trial compared to those who did not (median 4.0 [IQR 3.0, 6.0] and 5.0 [IQR 4.0, 6.0] respectively, *p* = 0.05).

**Table 2:** Rates of control for secondary prevention measures by study group

	Observation (***N*** = 71)		Control (***N*** = 22)		Intervention (***N*** = 16)	
	Baseline	12 months	Baseline	12 months	Baseline	12 months
**Antiplatelet use**	50/71 (70.4%)	51/71 (71.8%)	17/22 (77.3%)	15/22 (68.2%)	10/16 (62.5%)	10/16 (62.5%)
**Systolic BP (mmHg)**	147.8 (21.2)	152.1 (18.1)	148.3 (20.3)	152.4 (23.3)	143.7 (14.2)	156.1 (19.4)
**Diastolic BP (mmHg)**	80.3 (10.4)	84.5 (10.9)	80.2 (10.8)	81.1 (14.3)	82.7 (10.0)	88.9 (12.5)
**BP < 130/80 mmHg**	7/71 (9.9%)	2/71 (2.8%)	2/22 (9.1%)	1/22 (4.5%)	2/16 (12.5%)	0/16 (0.0%)
**BP < 140/90 mmHg**	24/71 (33.8%)	19/71 (26.8%)	7/22 (31.8%)	5/22 (22.7%)	6/16 (37.5%)	2/16 (12.5%)
**Total Cholesterol (mmol/L)**	4.9 (1.1)	4.4 (1.0)	4.9 (1.0)	4.3 (1.0)	4.1 (0.8)	3.9 (1.0)
**Total Cholesterol < 4.0 mmol/L**	16/71 (22.5%)	28/71 (39.4%)	4/22 (18.2%)	10/22 (45.5%)	8/16 (50.0%)	10/16 (62.5%)
**Heart rate (beats per min)**^**a,**^	75.7 (12.1)	74.5 (12.3)	68.4 (13.8)	72.3 (18.9)	78.3 (5.5)	71.1 (10.5)
**HR 60-80 bpm**^**a**^	10/21 (47.6%)	12/23 (52.2%)	2/3 (66.7%)	2/6 (33.3%)	3/5 (60.0%)	5/7 (71.4%)
**Adequate anticoagulation**^**a,b**^	8/21 (38.1%)	18/23 (78.3%)	3/3 (100.0%)	5/6 (83.3%)	1/5 (20.0%)	6/7 (85.7%)
**HbA1C mmol/mol**^c^	51.0 (44.3, 64.3)	49.0 (44.0, 69.3)	80.0 (−)	66.0 (−)	53.5 (−)	62.0 (−)
**HbA1C 48-53 mmol/mol**^c^	5/15 (33.3%)	4/17 (23.5%)	0/3 (0.0%)	0/3 (0.0%)	1/2 (50.0%)	1/3 (33.3%)

Average values and rates of control for secondary vascular prevention measures at baseline and 12 months by study group (restricted to participants who completed follow-up). Data presented are mean (SD), median (IQR), or frequency (%)

^a^Only those with AF

^b^INR 2.5–3.0 or on a DOAC

^c^Only those with diabetes

## Discussion

At present it is unclear whether control of multiple vascular risk factors can prevent further cognitive decline in vulnerable patients with a recent cerebrovascular event [5, 9, 18]. SERVED Memory aimed to test the feasibility of conducting such multimodal, guideline based, risk factor management in a pragmatic trial combining primary and secondary care input. We demonstrated a recruitment rate of > 20% of patients screened, suggesting that recruitment of patients with MCI associated with cerebrovascular disease to such a trial is possible. Although short of the recruitment target, the numbers entering the trial support its feasibility, especially given the proportion of patients with a MoCA score 20–25, or unknown at the point of screening, who declined to participate. However, nearly half of participants in the RCT arms did not complete follow-up, with this retention difficulty being partly related to frailty status. Alterations to the protocol may alleviate these difficulties, for example carrying out trial visits in the patients’ homes, using online assessments, or treatment changes being made directly by the trial team rather than relaying information to the GP. Such supported self-management strategies are deliverable in this patient population as evidenced by the TEST-BP trial [19], but these changes would inevitably increase the complexity and cost of conducting the trial. Additionally, although this trial was supported by a GP applicant, more involvement of primary care in future trial design would be valuable to explore why interventions were not being implemented.

In terms of the secondary objective of assessing the effect of the intervention we did not show a between-group difference in change in MoCA score over 12 months. Interestingly a greater decline in cognitive scores was seen in the observational cohort. These findings are in keeping with the results of two similar trials in patients with recent stroke but no early cognitive decline. Firstly, Ihle-Hansen et al. (*N* = 195) demonstrated no difference in incident cognitive impairment or dementia at 12 months with a multimodal intervention compared to usual GP care [20]. Secondly, Matz et al. (*N* = 202) reported no significant difference in cognitive test scores at 24 months between those treated with a multimodal vascular risk factor intervention and usual care [21]. Conversely, two larger trials in general populations have demonstrated that similar interventions can reduce cognitive decline and the risk of requiring long-term institutional care [22, 23]. Given the small sample size and short follow-up duration of all three existing studies in stroke patients, further trials may be warranted.

The main strength of this trial is the enrolment of patients with early cognitive decline, who are at increased risk of developing dementia this preventive strategy has not previously been assessed. A further strength is the use of a pragmatic real-world design, although this also served to highlight challenges in the optimisation of care for secondary stroke prevention that would need addressing in any future trial. A limitation is that we did not consult GP’s directly as to why targets were not being met, but it may reflect ongoing debate about the most appropriate risk factor targets (especially in older patients) [6, 7, 24], or excessive demands from the existing primary care workload. Assessment of the secondary trial objectives was also limited by the retention rate and small sample size. Furthermore, due to the lack of ethnic diversity in the trial population any findings may lack generalisability.

## Conclusions

Although the current protocol would not be feasible to deliver a definitive multi-centre trial due to difficulties with participant retention and application of the intervention, a successful further trial may be possible with protocol alterations as discussed. In addition, the findings of the epidemiological observation cohort suggest that such a trial should include patients with normal cognition and MCI following their cerebrovascular event, as all are at risk of further cognitive decline.

## Supplementary information

**Additional file 1: Table 1.** word document; “Rates of secondary prevention control at baseline in all participants”; average values and rates of control for secondary prevention measures at baseline by study group, including participants who did not complete the trial.

**Additional file 2: Table 2.** word document; “Rates of vascular risk factor treatment at baseline and 12 months”; rates of vascular risk factor treatment at baseline and 12 months by study group and changes during the trial (restricted to participants who completed follow-up).

**Additional file 3: Table 3.** word document; “Adverse events”; rates of serious adverse events, including deaths and recurrent stroke events, by study group.

## Data Availability

The datasets used and/or analysed during the current study are available from the corresponding author on reasonable request.

## Abbreviations

TIA: Transient ischaemic attack
MCI: Mild cognitive impairment
GP: General practitioner
BP: Blood pressure
AF: Atrial fibrillation
VaD: Vascular dementia
NNUH: Norfolk and Norwich University Hospital
MoCA: Montreal cognitive assessment
RCT: Randomised controlled trial
