# Supplementary material for: Can cardiovascular risk management be improved by shared care with general practice to prevent cognitive decline following stroke/TIA? A feasibility randomised controlled trial (SERVED memory)
Source: BMC Geriatr. 2020 Sep 17;20:353. doi: 10.1186/s12877-020-01760-z (PMC7499986; doi:10.1186/s12877-020-01760-z)
Supplement: Supplementary file 1 — Additional file 1: Table 1. word document; “Rates of secondary prevention control at baseline in all participants”; average values and rates of control for secondary prevention measures at baseline by study group, including participants who did not complete the trial. [file 12877_2020_1760_MOESM1_ESM.docx]

**Additional file 1:** Rates of secondary prevention control at baseline in all participants.

|  | **Observation (N=94)** | **Control (N=36)** | **Intervention (N=37)** |
| --- | --- | --- | --- |
| **Antiplatelet use** | 68/94 (72.3%) | 27/36 (75.0%) | 24/37 (64.9%) |
| **Systolic BP (mmHg)** | 147.3 (20.5) | 148.1 (21.0) | 145.2 (19.5) |
| **Diastolic BP (mmHg)** | 79.6 (10.5) | 78.9 (11.5) | 81.8 (12.5) |
| **BP <130/80mmHg** | 10/94 (10.6%) | 6/36 (16.7%) | 6/37 (16.2%) |
| **BP <140/90mmHg** | 35/94 (37.2%) | 12/36 (33.3%) | 15/37 (40.5%) |
| **Total Cholesterol (mmol/L)** | 4.9 (1.2) | 4.9 (1.2) | 4.6 (1.4) |
| **Total Cholesterol <4.0mmol/L** | 22/94 (23.4%) | 7/36 (19.4%) | 14/37 (37.8%) |
| **Heart rate (beats per min)^1,^** | 76.6 (18.9) | 75.9 (16.8) | 80.4 (10.2) |
| **HR 60-80bpm^1^** | 11/25 (44.0%) | 3/6 (50.0%) | 5/10 (50.0%) |
| **Adequate anticoagulation^1,2^** | 10/25 (40.0%) | 3/6 (50.0%) | 3/10 (30.0%) |
| **HbA1C mmol/mol^3^** | 52.5 (47.3, 69.5) | 49.5 (43.0, 82.3) | 73.0 (51.8, 106.3) |
| **HbA1C 48-53mmol/mol^3^** | 6/19 (31.6%) | 1/7 (14.3%) | 1/5 (20.0%) |

Average values and rates of control for secondary vascular prevention measures at baseline by study group (all participants). Data presented are mean (SD), median (IQR), or frequency (%).

^1^Only those with AF

^2^INR 2.5-3.0 or on a DOAC

^3^Only those with diabetes
