# Supplementary material for: Can cardiovascular risk management be improved by shared care with general practice to prevent cognitive decline following stroke/TIA? A feasibility randomised controlled trial (SERVED memory)
Source: BMC Geriatr. 2020 Sep 17;20:353. doi: 10.1186/s12877-020-01760-z (PMC7499986; doi:10.1186/s12877-020-01760-z)
Supplement: Supplementary file 2 — Additional file 2: Table 2. word document; “Rates of vascular risk factor treatment at baseline and 12 months”; rates of vascular risk factor treatment at baseline and 12 months by study group and changes during the trial (restricted to participants who completed follow-up). [file 12877_2020_1760_MOESM2_ESM.docx]

**Additional table 2:** Rates of vascular risk factor treatment at baseline and 12 months.

|  | | **Observation (N=71)** | | **Control (N=22)** | | **Intervention (N=16)** | |
| --- | --- | --- | --- | --- | --- | --- | --- |
|  |  | **Baseline** | **12 months** | **Baseline** | **12 months** | **Baseline** | **12 months** |
| **Antihypertensive medication (at least one agent)** | **Proportion treated** | 46/71  (64.8%) | 50/71  (70.4%) | 14/22 (63.6%) | 16/22  (72.7%) | 12/16  (75.0%) | 14/16  (87.5%) |
|  | **Treatment increased** | - | 12/71 (16.9%) | - | 8/22 (36.4%) | - | 6/16 (37.5%) |
|  | **Treatment decreased** | - | 12/71 (16.9%) | - | 1/22 (4.5%) | - | 2/16 (12.5%) |
|  | **Treatment unchanged** | - | 47/71 (66.2%) | - | 13/22 (59.1%) | - | 8/16 (50.0%) |
| **Statin or other lipid lowering medication** | **Proportion treated** | 52/71  (73.2%) | 56/71  (78.9%) | 20/22  (90.9%) | 20/22  (90.9%) | 9/16  (56.3%) | 10/16  (62.5%) |
|  | **Treatment increased** | - | 14/71 (19.7%) | - | 2/22 (9.1%) | - | 3/16 (18.7%) |
|  | **Treatment decreased** | - | 7/71 (9.9%) | - | 2/22 (9.1%) | - | 4/16 (25.0%) |
|  | **Treatment unchanged** | - | 50/71 (70.4%) | - | 18/22 (81.8%) | - | 9/16 (56.3%) |
| **Rate lowering medication (e.g. beta blocker)** | **Proportion treated** | 10/21  (47.6%) | 11/23  (47.8%) | 0/3  (0.0%) | 4/6  (66.7%) | 3/5  (60.0%) | 5/7  (71.4%) |
|  | **Treatment increased** | - | 0/23 (0.0%) | - | 4/6 (66.6%) | - | 3/7 (42.9%) |
|  | **Treatment decreased** | - | 2/23 (8.7%) | - | 0/6 (0.0%) | - | 0/7 (0.0%) |
|  | **Treatment unchanged** | - | 21/23 (91.3%) | - | 2/6 (33.4%) | - | 4/7 (57.1%) |
| **Warfarin or direct oral anticoagulant** | **Proportion treated** | 13/21  (61.9%) | 19/23  (82.6%) | 3/3  (100.0%) | 5/6 (83.3%) | 3/5  (60.0%) | 6/7 (85.7%) |
|  | **Treatment increased** | - | 8/23 (34.8%) | - | 2/6 (33.4%) | - | 3/7 (42.9%) |
|  | **Treatment decreased** | - | 2/23 (8.7%) | - | 0/6 (0.0%) | - | 1/7 (14.2%) |
|  | **Treatment unchanged** | - | 13/23 (56.5%) | - | 4/6 (66.6%) | - | 3/7 (42.9%) |
| **Oral diabetic medications or insulin** | **Proportion treated** | 8/15  (53.3%) | 8/17  (47.1%) | 2/3  (66.7%) | 2/3  (66.7%) | 2/2  (100.0%) | 3/3  (100.0%) |
|  | **Treatment increased** | - | 0/17 (0.0%) | - | 0/3 (0.0%) | - | 1/3 (33.4%) |
|  | **Treatment decreased** | - | 0/17 (0.0%) | - | 0/3 (0.0%) | - | 0/3 (0.0%) |
|  | **Treatment unchanged** | - | 17/17 (100.0%) | - | 3/3 (100.0%) | - | 2/3 (66.6%) |

Rates of vascular risk factor treatment at baseline and 12 months by study group and changes during the trial (restricted to participants who completed follow-up). Data presented are frequency (%).
