# Supplementary material for: Can cardiovascular risk management be improved by shared care with general practice to prevent cognitive decline following stroke/TIA? A feasibility randomised controlled trial (SERVED memory)
Source: BMC Geriatr. 2020 Sep 17;20:353. doi: 10.1186/s12877-020-01760-z (PMC7499986; doi:10.1186/s12877-020-01760-z)
Supplement: Supplementary file 3 — Additional file 3: Table 3. word document; “Adverse events”; rates of serious adverse events, including deaths and recurrent stroke events, by study group. [file 12877_2020_1760_MOESM3_ESM.docx]

**Additional table 3:** Adverse events.

|  | **Observation** | **Control** | **Intervention** |
| --- | --- | --- | --- |
| Serious adverse events | 36 | 25 | 24 |
| Deaths | 3 | 1 | 2 |
| Recurrent stroke/TIA events | 7 | 2 | 5 |
| Withdrawals due to ill health (other than recurrent stroke/TIA) | 2 | 2 | 0 |

Rates of serious adverse events, including deaths and recurrent stroke events, by study group.
